# Liver X receptor agonist T0901317 reverses resistance of A549 human lung cancer cells to EGFR‐TKI treatment

**DOI:** 10.1002/2211-5463.12147

**Published:** 2016-12-20

**Authors:** Haixia Cao, Shaorong Yu, Dan Chen, Changwen Jing, Zhuo Wang, Rong Ma, Siwen Liu, Jie Ni, Jifeng Feng, Jianzhong Wu

**Affiliations:** ^1^ Research Center for Clinical OncologyJiangsu Cancer Hospital and Jiangsu Institute of Cancer ResearchNanjing Medical University Affiliated Cancer HospitalJiangsu ProvinceChina; ^2^ Department of OncologyJiangsu Cancer Hospital and Jiangsu Institute of Cancer ResearchNanjing Medical University Affiliated Cancer HospitalJiangsu ProvinceChina; ^3^ The Fourth Clinical School of Nanjing Medical UniversityJiangsu ProvinceChina

**Keywords:** EGFR‐TKI, liver X receptors, lung cancer, resistance

## Abstract

Epidermal growth factor receptor‐tyrosine kinase inhibitor (EGFR‐TKI) is effective in lung cancer patients carrying sensitive EGFR mutations. In this study, we investigated if liver X receptor (LXR) agonist T0901317 could reverse the resistance of lung cancer cell lines A549 and H1650 to EGFR‐TKI treatment. We found that T0901317 could make natural EGFR‐TKI‐resistant A549 human lung cancer cells sensitive to EGFR‐TKI treatment and that this was dependent on LXRβ expression. However, T0901317 does not have a similar effect on another natural EGFR‐TKI‐resistant cell line H1650.

**Abbreviations**
CCK‐8: Cell Counting Kit‐8
EGFR‐TKI: epidermal growth factor receptor‐tyrosine kinase inhibitor
LXR: liver X receptor
MAPK: mitogen‐activated protein kinase
PDK1: phosphoinositide‐dependent protein kinase‐1
PI3K: phosphoinositide 3‐kinase
siRNA: small‐interference RNA
SI: synergistic indices

Lung cancer is the most common cancer in the world and causes the leading cancer‐related death worldwide. Approximately 80% of lung cancers are non‐small cell lung cancer (NSCLC) and most of these newly diagnosed cancer patients have advanced‐stage disease and lost the opportunity of surgical therapy.

Several large clinical studies have proved that epidermal growth factor receptor‐tyrosine kinase inhibitor (EGFR‐TKI) is effective in NSCLC patients carrying sensitive EGFR mutations 1, 2, 3, 4. EGFR‐TKI has been regarded as the standard first‐line treatment in advanced NSCLC patients with sensitive EGFR mutations. However, acquired EGFR‐TKI resistance will eventually develop in most patients within 12 months 1, 5, 6. Although novel compounds designed to act on targets associated with EGFR‐TKI resistance have been in continued clinical development, how to overcome EGFR‐TKI resistance still needs further research.

Liver X receptors (LXRs) are members of the nuclear receptor family of ligand‐dependent transcription factors and are well characterized as regulators of cholesterol, glucose, and fatty acid metabolism and inflammatory responses 7, 8, 9. Natural and synthetic ligands have been developed for the treatment of metabolic and inflammatory conditions and diseases. LXR agonists can disrupt the proliferation of several cell types via altering expression levels of genes involved in controlling cell cycle and reduce angiogenesis in pathological conditions of uncontrolled angiogenesis 10, 11, 12, 13, 14. Recently, accumulating evidence indicates the important functional role of LXRs in a variety of malignancies, affecting both tumor growth and tumor metastasis 15, 16, 17, 18. For example, the synthetic ligand T0901317 can inhibit prostate cancer cell proliferation and tumor formation and overexpression of LXRα can sensitize cancer cells to the ligand 13.

Epidermal growth factor receptor mutations and phosphoinositide 3‐kinase (PI3K) hyperactivation are common in glioblastoma. Therefore, glioblastoma is naturally resistant to EGFR‐TKI treatment 19. In this tumor model, researchers found that the LXR agonist can promote glioblastoma cell death through inhibition of an EGFR/Atk/SREBP‐1/LDLR‐dependent pathway 20. Prostate cancer cells are often resistant to standard chemotherapeutic options due to their consistent activation of Akt. However, treatment with the LXR agonist can donwnregulate the AKT survival pathway and induce apoptosis of prostate cancer cells in both xenografted nude mice and cell culture 21. Previous research shows that LXR agonist can suppress the NF‐κB pathway 22, 23. All these lines of research together showed that LXR agonist could suppress the EGFR‐PI3K‐AKT‐NF‐κB pathway. Thus, we hypothesized that the LXR agonist might sensitize EGFR‐TKI‐resistant human lung cancer cells to EGFR‐TKI treatment by suppressing the EGFR‐PI3K‐AKT‐NF‐κB pathway.

Wairagu *et al*. proved that LXR had combined therapeutic potential with EGFR‐TKI in lung cancer cells 24. Their results found that the combined treatment with EGFR inhibitor gefitinib and T0901317 showed additive growth inhibition by inhibiting cyclinD1 and cyclinB expression in both H2073 and H1993 cells. However, the precise mechanisms are still unknown. We previously demonstrated that the LXR agonist can sensitize the acquired EGFR‐TKI resistant human lung cancer cells HCC827 by inhibiting Akt activation 25. The aim of this study was to investigate whether the synthetic LXR agonist T0901317 can reverse EGFR‐TKI resistance of lung cancer cell lines A549 and H1650 and try to indentify its potential mechanism.

## Materials and methods

### Cell line

H1650 and A549 were purchased from Shanghai Institutes for Biological Sciences, Chinese Academy of Cell Resource Center (Shanghai, China). H1650 is a lung adenocarcinoma cell line with co‐occurrence of an EGFR mutation (in‐frame deletion in exon 19) and homozygous deletion of PTEN. A549 is a NSCLC line carrying the KRAS mutation. Cells were cultured at 37 °C with 5% CO_2_ in RPMI 1640 supplemented with 10% heat‐inactivated FBS, 100 units·mL^−1^ penicillin and 100 units·mL^−1^ streptomycin. Both H1650 and A549 cells have been proved to be naturally resistant to gefitinib. The IC_50_ for the typical gefitinib‐sensitive lung cancer cell HCC827 was 0.052 ± 0.024 μm whereas the IC_50_ for both H1650 and A549 is > 10 μm.

### Reagents and antibodies

T0901317 was purchased from Cayman Chemical (Ann Arbor, MI, USA) and gefitinib (Iressa) from AstraZeneca UK Limited (Macclesfield, Cheshire, UK). Stock solutions of all drugs were prepared in DMSO and stored at −20 °C. Antibodies against caspase‐9 (9502), caspase‐3 (9665), cleaved caspase‐3 (9664), cleaved caspase‐9 (9501), Bax (5023), AKT (4691), p‐Akt (Ser473) (4060), PDK1 (3062), p‐PDK1 (Ser241) (3438), PI3 kinase p85 (4257), p‐PI3 kinase p85 (Tyr458)/p55 (Tyr199) (4228), 44/42 MAPK (4695), p‐44/42 MAPK (Thr202/Tyr204) (4370), p‐EGFR (Tyr1173) (4407), cyclin D1 (2978), and LXRβ (13519) were obtained from Cell Signaling Technology (Danvers, MA, USA). Antibodies against LXR‐α (PP‐K8607‐00) were purchased from Perseus Proteomics (Tokyo, Japan); antibodies against β‐actin from Bioword Technology (Louis Park, MN, USA) and antibodies against EGFR (ab47479) from Abcam (Cambridge, MA, USA).

### Cell viability assay

Cell viability was measured using the CCK‐8 assay. Briefly, cells were seeded in 96‐well plates and were allowed to settle. After starvation for 6 h, cells were treated with different conditions of drugs and continuously exposed to each drug for 4 days. Each assay was set up in six replicate wells. Control values of untreated cells were taken as 100%, and viability data of treated ones were expressed as percentage of controls.

### Quantitative real‐time PCR (qRT‐PCR)

Total RNAs of cells treated with T0901317 were extracted using the Trizol reagent (Invitrogen, Carlsbad, CA, USA). Qualified RNA was used for cDNA synthesis with the PrimeScript® RT reagent (Takara Biotechnology, Dalian, China) according to the manufacturer's instructions. The real‐time PCR was performed using SYBR Select Master Mix (Life Technologies, Austin, TX, USA) on a 7300 real‐time PCR system (Applied Biosystems, Foster City, CA, USA). The cycling conditions were set by the manufacturer's protocol. Primers of targeted genes and β‐actin were similar to those used previously 24. The ΔΔ cycle threshold (2^−ΔΔCt^) method was used to determine the fold change of LXRα and LXRβ. Expression data were normalized by β‐actin.

### Colony formation assay

A549 cells were seeded in six‐well plates at a density of 200 cells per well. After treatment with gefitinib (1 μm) and T0901317 (1 μm) alone or in combination for 6 days, the cells were allowed to incubate in RPMI 1640 at 37 °C for 1 week. Cells were then fixed and stained with 0.5% crystal violet solution for 10 min and photographed. The colony forming efficiency was determined by the percentage of colonies consisting of 50 or more cells.

### Small‐interference RNA transfection

Small‐interference RNA (siRNA) duplexes for LXRα and LXRβ were designed and synthesized by RiboBio Co., Ltd. (Guangzhou, China). A549 cells were transfected with siRNA or negative control using Lipofectamine 2000 (Invitrogen) according to the manufacturer's protocol.

### Evaluation of cell apoptosis

To quantify cell apoptosis, annexin V/PI staining was performed. Briefly, after treatment with gefitinib (5 μm) and T0901317(5 μm) alone or in combination for 4 days, both floating and attached cells were collected and subjected to annexin V/PI staining using an FITC Annexin V Apoptosis Detection Kit (BD Biosciences, San Diego, CA, USA) according to the manufacturer's protocol. Analysis by BD Accuri C6 flow cytometer (Becton Dickinson, San Diego, CA, USA) was carried out to discriminate apoptotic cells. At least 10 000 cells were analyzed by FACS for each cell population.

### Flow cytometric analysis of cell cycle distribution

After appropriate treatment, cells were washed twice with ice‐cold phosphate‐buffered saline (PBS), harvested, and fixed in cool 70% ethanol at 4 °C overnight. After fixation, cells were incubated with 50 mg·mL^−1^ PI staining solution with RNase A for 30 min at room temperature. The distribution of different DNA contents was analyzed by a FACScan flow cytometer (Becton Dickinson).

### Western blot

Cells were lyzed in RIPA buffer (Beyotime Biotechnology, Jiangsu, China). Lysates were centrifuged at 14 000 ***g*** for 15 min at 4 °C. The supernatant was used for subsequent procedures. After boiling in a NuPAGE LDS sample buffer (Life Technologies), samples were subjected to NuPAGE 10% Bis–Tris gel (Life Technologies) and then transferred onto polyvinylidene difluoride membranes (Millipore Corporation, Billerica, MA, USA). Membranes were incubated overnight at 4 °C with a primary antibody, incubated for 1 h with a HRP‐conjugated secondary antibody, and visualized using the ECL Plus Kit (Beyotime Biotechnology).

### Statistical analysis

Each experiment was repeated at least three times. All values are expressed as mean ± SD. QPCR data were analyzed using the unpaired, two‐sided Student's *t* test with microsoft excel 2010. Other data were analyzed using one‐way ANOVA with graphpad 5.01. Statistical difference was considered significant when *P* < 0.05.

## Results

### Effects of T0901317 on A549 and H1650 cells

**Figure 1:**
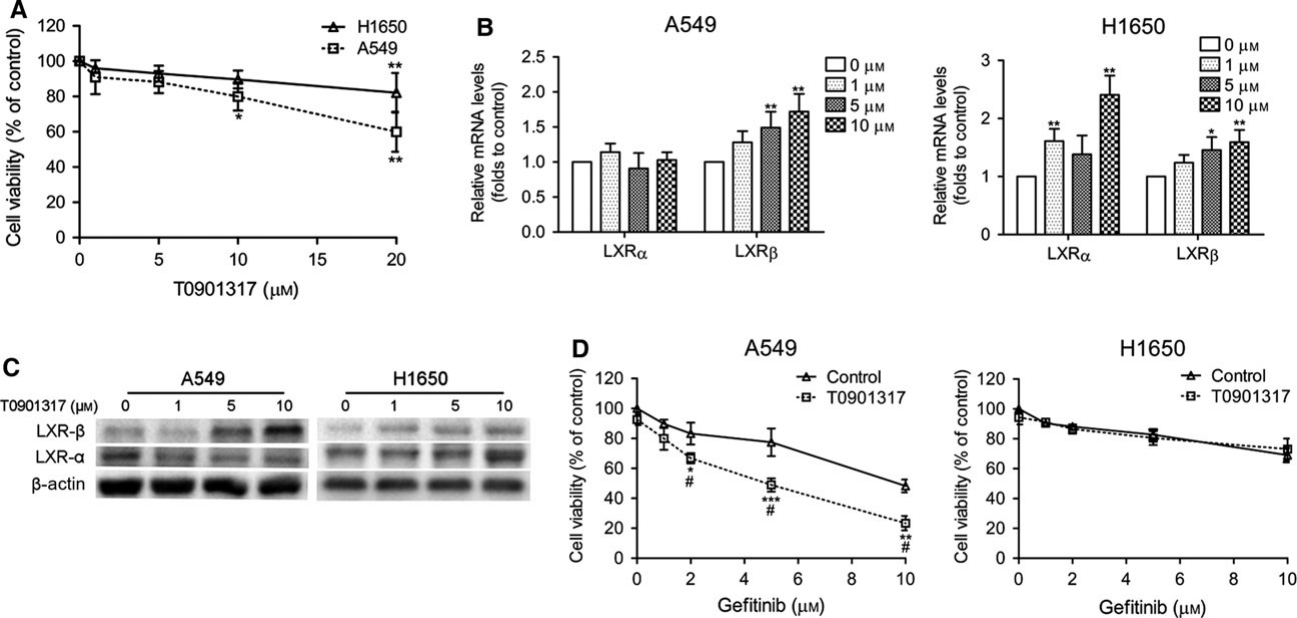
Effects of T0901317 on A549 and H1650 cells. (A) CCK8 assay was performed after treatment of cells with increasing concentrations of T0901317 for 4 days. Cell viabilities are presented as percentages of the values in the untreated groups. *P < 0.01, **P < 0.001 versus untreated groups. (B) mRNA expression levels of LXRα and LXRβ were measured using the QPCR assay. *P < 0.05, **P < 0.01. (C) LXRα and LXRβ protein expressions were assayed using western blot analysis. (D) Cells were treated with increasing concentrations of gefitinib alone or in combination with 5 μm TO901317 for 4 days. Growth was assessed by the CCK8 assay. *P < 0.1, **P < 0.01, ***P < 0.001 versus gefitinib control groups, ^#^ *P* < 0.001 versus T0901317 control groups.

We first examined the cytotoxicity of T0901317 in A549 and H1650 cells. As shown in Fig. 1A, treatment with T0901317 slightly decreased the viability of H1650 cells in a dose‐dependent manner and the response by A549 at significantly lower concentrations of the drug than H1650. The expression of LXRα and LXRβ was then analyzed after treatment with T0901317 (Fig. 1B,C). The expression of LXRβ slightly increased at higher doses of T0901317 (5 and 10 μm) in both A549 and H1650 cells and the expression of LXRα increased in H1650 cells while it did not exhibit any difference in A549 cells. Thereafter, we chose 5 μm T0901317 in combination with increasing concentrations of gefitinib (1–10 μm) to investigate their effects on cells (Fig. 1D). Interestingly, the combined treatment of T0901317 with gefitinib showed more additive growth inhibitory response when compared with the drug alone in A549 cells, but there was no significant difference in H1650 cells.

### T0901317 sensitizes gefitinib by inducing apoptosis and cell cycle arrest in A549 cells

**Figure 2:**
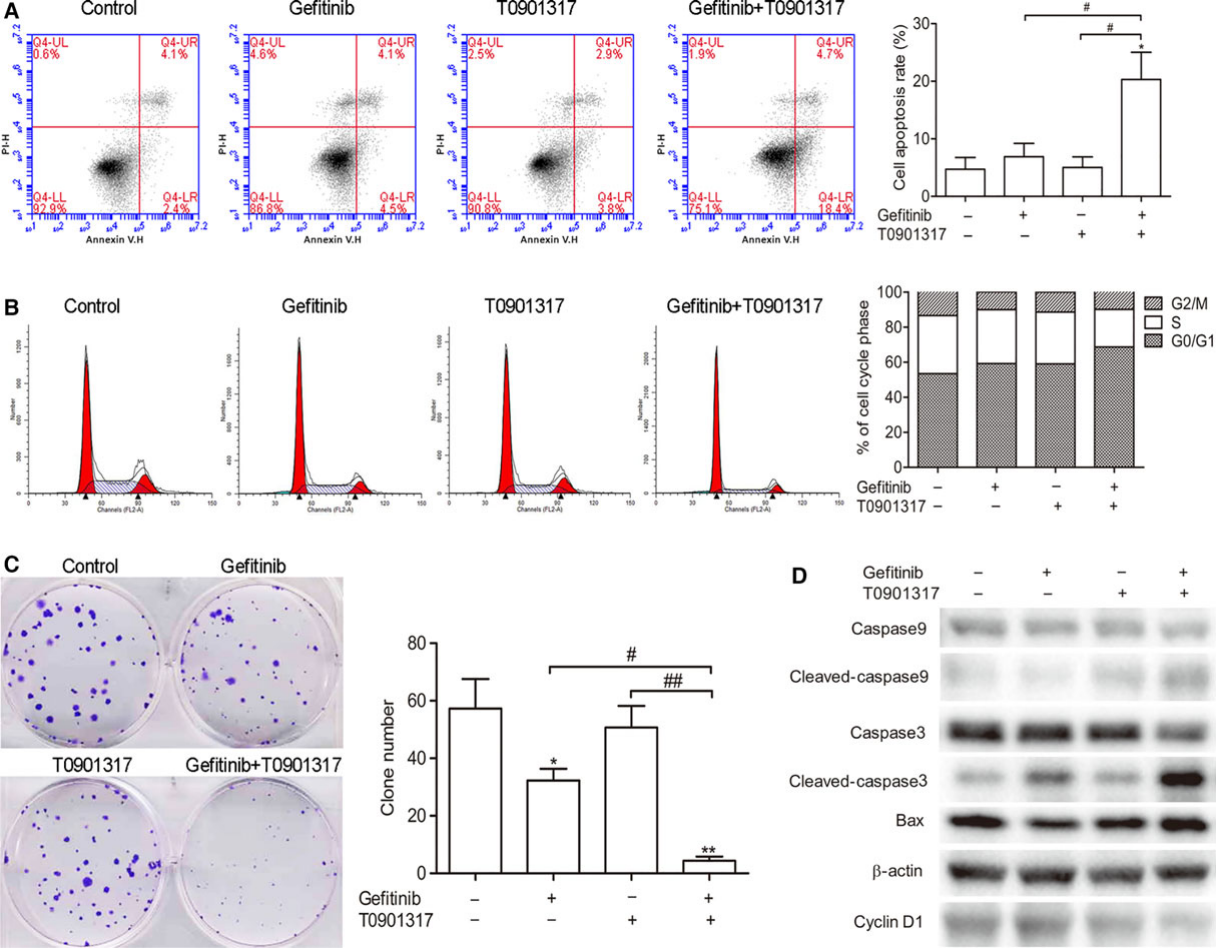
T0901317 sensitizes gefitinib by inducing apoptosis and cell cycle arrest in A549 cells. After treatment with gefitinib (5 μm) and/or T0901317 (5 μm) for 4 days, cell cycle and cell apoptosis were analyzed. (A) Cell apoptosis was analyzed by flow cytometer with annexin V/PI staining. *P < 0.001 versus untreated groups; ^#^ *P* < 0.001 between two groups. (B) Flow cytometry analysis of cell cycle distribution. (C) Colony formation assays were performed in A549 cells. *P < 0.01, **P < 0.001 versus untreated groups; ^#^ *P* < 0.01, ^##^ *P* < 0.001 between two groups. (D) Western blot analyses were performed using the indicated antibodies.

To better understand the mechanics of the synergistic effects of T0901317 on gefitinib in A549 cells, cell apoptosis and cycle analysis were performed following treatment. Flow cytometry analysis revealed that the percentage of apoptosis induced by the combined treatment was dramatically increased when compared with T0901317 or gefitinib alone (Fig. 2A). Cells in the G1/G0 phases of the cell cycle increased in number upon combined treatment whereas a corresponding decrease in cells in the S phase was observed (Fig. 2B). These changes are statistically significant. Western blot analysis showed that combination therapy increased the expression of cleaved caspase‐3, cleaved caspase‐9, and Bax, and decreased the expression of cyclin D1 (Fig. 2D). Clonogenic assays were also employed to evaluate the effects of long‐term treatment on cell proliferation and colony formation (Fig. 2C). The results showed that gefitinib alone inhibited colony formation and the combination therapy further augmented this effect. These results indicate that induction of apoptosis and cell cycle arrest may be an important mechanism underlying the synergistic effects of T0901317 on gefitinib in A549 cells.

### T0901317 sensitizes gefitinib by reducing the phosphorylation of AKT

**Figure 3:**
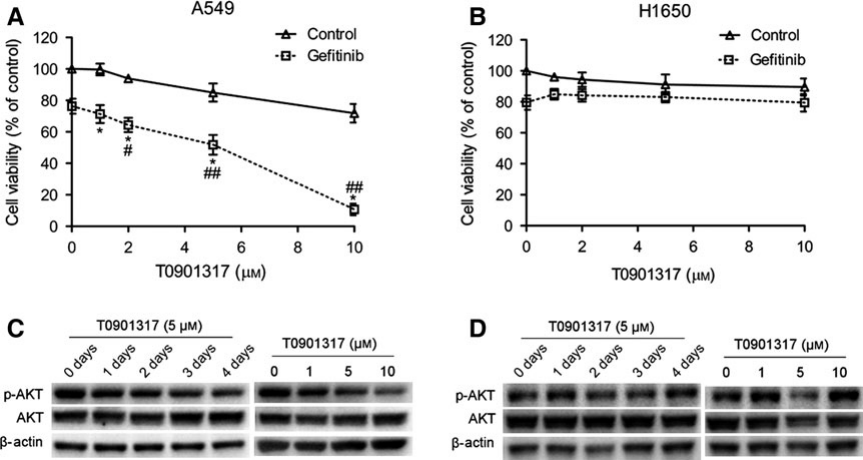
T0901317 sensitizes gefitinib by inhibiting AKT activation in A549 cells. (A, B) CCK8 assay was performed after A549 and H1650 cells were treated with increasing concentrations of TO901317 alone or in combination with 5 μm gefitinib. *P < 0.001 versus T0901317 control groups, ^#^ *P* < 0.01, ^##^ *P* < 0.001 versus gefitinib control groups. (C) Western blot analysis was performed to detect the expression of AKT and p‐AKT after knockdown of LXRα and LXRβ expression. (D) After knockdown of LXR expression, A549 cells were treated with 5 μm T0901317 for 4 days. The expression of AKT and p‐AKT was detected by western blot analysis.

We chose 5 μm gefitinib combined with increasing concentrations of T0901317 (1–10 μm) to investigate the effect of T0901317. Compared with gefitinib alone, T0901317 resensitizes gefitinib in a dose‐dependent manner in A549 cells (Fig. 3A) while it had no significant effect on gefitinib sensitivity in H1650 cells (Fig. 3B).

Our previous study showed that LXR ligands sensitize gefitinib by inhibiting AKT activation in gefitinib‐resistant HCC827 cells. This mechanism might have caused different effects of T0901317 on gefitinib in A549 and H1650 cells. As expected, the phosphorylation of AKT is reduced time and dose dependently with T0901317 in A549 cells (Fig. 3C). When it comes to H1650 cells, the phosphorylation of AKT was not decreased but increased after treatment with different concentrations of T0901317 for 4 days (Fig. 3D).

### Effect of LXR knockdown on the synergistic effects of T0901317 and gefitinib in A549 cells

**Figure 4:**
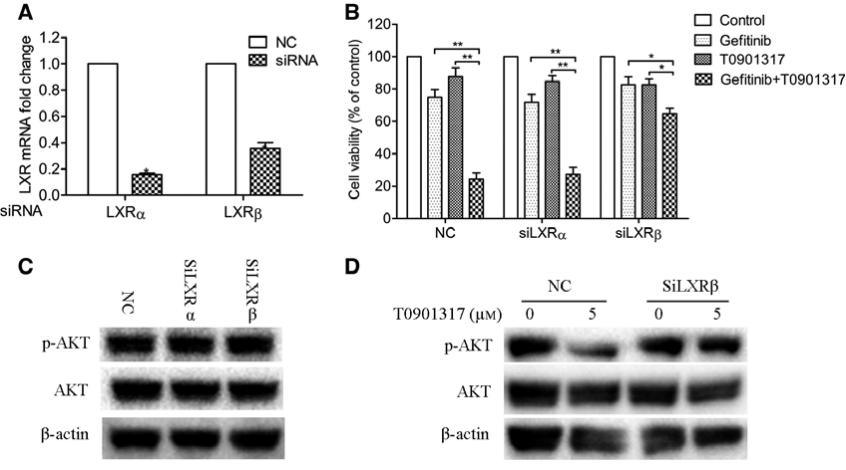
Knockdown of LXRβ expression blocks the synergistic effects of T0901317 on gefitinib in A549 cells. (A) Knockdown of LXRα and LXRβ expression was validated by quantitative PCR. *P < 0.001 versus NC groups. (B) The effect of LXR knockdown on A549 cell proliferation was quantified by CCK8 assays. Cells were treated with 5 μm gefitinib alone or in combination with 5 μm T0901317 for 4 days. *P < 0.01, **P < 0.001 between two groups. (C, D) Western blot analysis was performed to detect the expression of AKT and p‐AKT after A549 (C) and H1650 (D) cells were treated with 5 μm T0901317 for different days or increasing concentrations of T0901317 for 4 days.

To determine the role of LXRs in mediating the effects of the ligands, we knocked down LXRα and LXRβ expression using small interfering RNAs (siRNAs). Compared to the controls, LXRα and LXRβ expressions were reduced by 15.7% and 35.6% respectively when A549 cells were transfected with the corresponding siRNA (Fig. 4A). After knockdown of LXRs, the combined treatment of T0901317 and gefitinib still showed a significantly additive growth inhibitory response when compared to drugs alone (Fig. 4B). However, this response was reduced in the LXRβ knockdown group. Synergistic indices of the combination treatment with T0901317 and gefitinib showed that the LXRβ knockdown group had a slightly synergistic effect (SI = 1.06) while the LXRα knockdown group (SI = 2.26) and negative control group (SI = 2.75) showed a significant effect (Table 1). These results indicate that LXRβ is required for A549 cells in the synergistic effects of T0901317 on gefitinib treatment.

**Table 1:** Synergistic indices of combination treatment with gefitinib and T0901317 after knockdown of LXRα or LXRβ expression. MGI, mean growth inhibition rate = growth rate of treated group/growth rate of untreated group. SI, synergistic index = expected growth inhibition rate/observed growth inhibition rate. An index of more than 1 indicates synergistic effect and < 1 indicates a less than additive effect

siRNA	Gefitinib		T0901317		Combination			SI
	MGI	*P* a	MGI	*P* a	Expected^b^	Observed^c^	*P* a	
NC	0.75	< 0.001	0.88	> 0.05	0.66	0.24	< 0.001	2.75
siLXRα	0.72	< 0.001	0.85	< 0.05	0.61	0.27	< 0.001	2.26
siLXRβ	0.83	< 0.05	0.83	< 0.05	0.69	0.65	< 0.001	1.06

a *P* value (two‐sided) was calculated by one‐way ANOVA compared with no treatment.

b Expected growth inhibition rate = MGI T0901317 × MGI gefitinib.

c Observed growth inhibition rate = growth inhibition rate of combined treatment/growth rate of untreated group.

Western blot analysis showed that AKT phosphorylation was slightly increased after knockdown of LXRβ (Fig. 4C). After treatment with T0901317 for 4 days, AKT phosphorylation was dramatically reduced in control conditions. However, it was slightly decreased in knockdown of LXRβ conditions (Fig. 4D). Taken together, these results show that AKT inactivation was mediated by LXRβ activation in A549 cells.

### T0901317 sensitizes gefitinib by inhibiting the PI3K/Akt signal pathway in A549 cells

**Figure 5:**
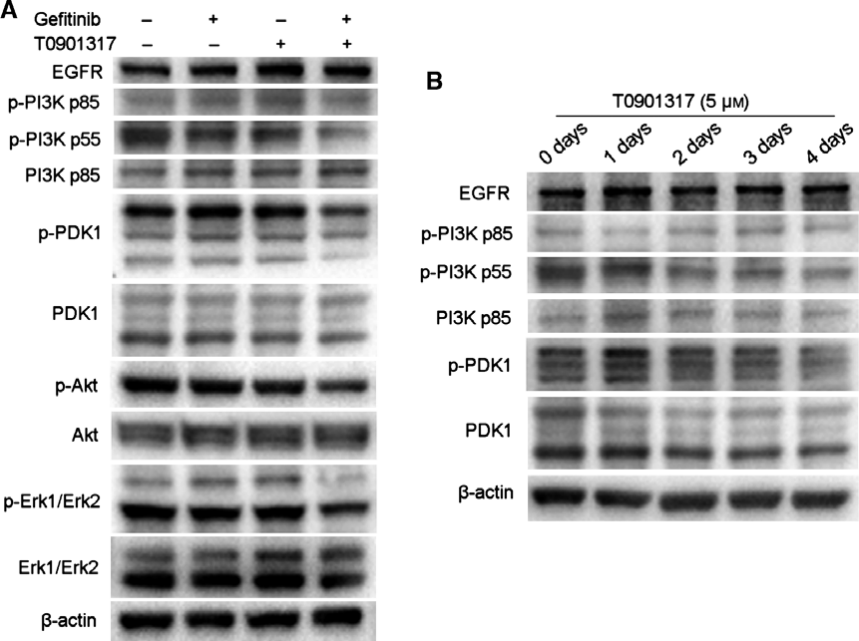
T0901317 sensitizes gefitinib by inhibiting the PI3K/Akt signal pathway. (A) A549 cells were treated for 3 days with gefitinib (5 μm) alone, T0901317 (5 μm) alone, or the two drugs in combination. Cells were lyzed and the indicated proteins were detected by western blot. (B) Western blot analysis was performed using the indicated antibodies after A549 cells were treated with 5 μm T0901317 for different days.

To further uncover potential mechanisms of the effect of T0901317 on gefitinib in A549 cells, we detected protein expression of the PI3K/Akt signal pathway and EGFR. First, cells were treated with T0901317, gefitinib, or their combination. Phosphorylation of PI3K p55, PDK1, and Akt was substantially reduced by the combination of gefitinib and T0901317 (Fig. 5A). To further assess the downstream effects of a downregulated PI3K/Akt signal pathway, ERK (p44/p42) and phosphorylation status were determined. Decreased phospho‐ERK level was also observed in the combined treatment group. These findings suggest that combined treatment could cause a change in cells by inhibiting the PI3K/Akt signal pathway.

We then examined gene expression of the PI3K/Akt signal pathway after treatment with T0901317. The results showed that phosphorylation of PI3K p55 decreased from day 2 and phosphorylation of PDK1 decreased only after treatment for 4 days (Fig. 5B). These results indicate that T0901317 could inhibit the PI3K/Akt signal pathway, and the combination treatment with gefitinib further augmented this inhibition.

## Discussion

In this study, we showed that the LXR agonist T0901317 could sensitize natural EGFR‐TKI‐resistant human lung cancer cell A549 to EGFR‐TKI. However, T0901317 has no similar effect on another natural EGFR‐TKI‐resistant cell line H1650. We proved that the resensitization of A549 was achieved by inhibiting Akt pathway activation dependent on LXR β expression.

Although LXR was reported to have antiproliferative effects on cancer cells 16, 26, we showed that there was no cytotoxicity of T0901317 in lung cancer cells in low concentrations (< 10 μm). Therefore, we chose 5 μm of T0901317 for further research. Combined treatment of different concentrations of gefitinib and 5 μm of T0901317 showed more than an additive growth inhibitory response when compared with the drug alone in A549 cells. However, there was no significant difference in H1650 cells. Increasing concentrations of T0901317 combined with 5 μm gefitinib did not inhibit H1650 cell viability, implying that T0901317 could not sensitize H1650 to gefitinib. Therefore, we chose A549 for further research.

We found that T0901317 sensitized gefitinib in A549 cells by inducing apoptosis, cell cycle arrest and inhibiting colony formation of A549 cells. The pro‐apoptotic effect was achieved through the caspase‐3 pathway. We found that the phosphorylation of Akt reduced time and dose dependently with T0901317 treatment in A549 cells; however, the phosphorylation of Akt did not decrease after treatment of H1650 cells with T0901317. This result proved that T0901317 alone can inhibit the activation of Akt by reducing the phosphorylation of Akt in A549 cells. That T0901317 could not sensitize gefitinib in H1650 cells could be attributed to its failure to inhibit the activation of Akt in H1650 cells.

To further investigate whether T0901317 can suppress key signal molecules of the PI3K/Akt pathway, we detected the protein expression of EGFR and the PI3K/Akt signal pathway. We found that the phosphorylation of PI3K p55 and PDK1 decreased after treatment with T0901317 alone. The phosphorylation of PI3K p55, PDK1, Akt, and ERK (p44/p42) was substantially reduced after the combination treatment with gefitinib and T0901317, suggesting possible inhibition of the PI3k/Akt pathway. These results support our hypothesis that T0901317 sensitizes A549 to EGFR‐TKI by suppressing the EGFR/PI3K/Akt signal pathway. The LXR agonist can inhibit activation of the EGFR‐PI3K‐AKT‐NF‐κB pathway and has a potential synergistic effect with EGFR‐TKI treatment 20, 21, 22, 24. Our results prove this hypothesis and confirmed the inhibitory effect of the LXR agonist on the Akt pathway in A549 cells.

There are two isoforms of LXRs, LXRα and LXRβ. LXRα is highly expressed in the prostate, breast, pancreas, colon, and liver, whereas LXRβ is ubiquitously expressed 27. Despite similar characteristics such as high sequence homology and a similar ligand profile, they have distinct and specific functions. The LXRβ‐dependent pathway can induce colon cancer cell pyroptosis 28. However, LXR could be synergistic in human carcinomas because of signaling interactions mediated through LXRα 29. We detected the expression of LXRα and LXRβ in A549 cells after treatment with T0901317 and found that the expression of LXRβ increased at a higher dose of T0901317, suggesting that T0901317 may sensitize A549 cells to gefitinib through LXRβ. To confirm this hypothesis, we knocked down the expression of LXRα and LXRβ. The synergistic effect disappeared after LXRβ knockout (SI: 1.06 vs. 2.75, *P* < 0.001) whereas the synergistic effect did not change after LXRα knockout (SI: 2.26 vs. 2.75). Thus, we confirm that the synergistic effect was achieved by LXRβ expression rather than LXRα expression.

In summary, our study demonstrated that the LXR agonist T0901317 sensitizes EGFR‐TKI‐resistant human lung cancer cell A549 to EGFR‐TKI treatment *in vitro*, and this effect was partly achieved by inhibition of the PI3K/Akt signaling pathway. The synergistic effect of the LXR agonist in a nude mice model will be explored in more detail in a future study.

## Author contributions

JF and JW conceived and supervised the experiment and modified the manuscript. HC and SY participated in the design of this study, carried out the western blot experiment, and drafted the manuscript. DC, CJ, and ZW carried out the cell and RT‐PCR experiments. RM and SL conducted small‐interference RNA transfection experiments. JN performed the statistical analysis. All authors read and approved the final manuscript.
